# Insight into *S*-adenosylmethionine biosynthesis from the crystal structures of the human methionine adenosyltransferase catalytic and regulatory subunits

**DOI:** 10.1042/BJ20121580

**Published:** 2013-04-25

**Authors:** Naeem Shafqat, Joao R. C. Muniz, Ewa S. Pilka, Evangelos Papagrigoriou, Frank vonDelft, Udo Oppermann, Wyatt W. Yue

**Affiliations:** *Structural Genomics Consortium, University of Oxford, Oxford OX3 7DQ, U.K.; †Botnar Research Centre, NIHR Oxford Biomedical Research Unit, University of Oxford, Oxford OX3 7LD, U.K.

**Keywords:** methionine adenosyltransferase, methionine adenosyltransferase 1A deficiency (MAT1A deficiency), *S*-adenosylmethionine, short-chain dehydrogenase/reductase, AMP-PNP, adenosine 5′-[β,γ-imido]triphosphate, DSF, differential scanning fluorimetry, eMAT, *Escherichia coli* methionine adenosyltransferase, HCC, hepatocellular carcinoma, hMAT, human MAT, MAT, methionine adenosyltransferase, Met, L-methionine, NCS, non-crystallographic symmetry, P_i_, orthophosphate, PP_i_, pyrophosphate, PPP_i_, tripolyphosphate, rMAT, rat MAT, RMSD, root mean square deviation, SAM, *S*-adenosylmethionine, SDR, short-chain dehydrogenase/reductase, TCEP, tris-(2-carboxyethyl)phosphine, TEV, tobacco etch virus, TMAO, trimethylamine *N*-oxide

## Abstract

MAT (methionine adenosyltransferase) utilizes L-methionine and ATP to form SAM (*S*-adenosylmethionine), the principal methyl donor in biological methylation. Mammals encode a liver-specific isoenzyme, MAT1A, that is genetically linked with an inborn metabolic disorder of hypermethioninaemia, as well as a ubiquitously expressed isoenzyme, MAT2A, whose enzymatic activity is regulated by an associated subunit MAT2B. To understand the molecular mechanism of MAT functions and interactions, we have crystallized the ligand-bound complexes of human MAT1A, MAT2A and MAT2B. The structures of MAT1A and MAT2A in binary complexes with their product SAM allow for a comparison with the *Escherichia coli* and rat structures. This facilitates the understanding of the different substrate or product conformations, mediated by the neighbouring gating loop, which can be accommodated by the compact active site during catalysis. The structure of MAT2B reveals an SDR (short-chain dehydrogenase/reductase) core with specificity for the NADP/H cofactor, and harbours the SDR catalytic triad (YxxxKS). Extended from the MAT2B core is a second domain with homology with an SDR sub-family that binds nucleotide-sugar substrates, although the equivalent region in MAT2B presents a more open and extended surface which may endow a different ligand/protein-binding capability. Together, the results of the present study provide a framework to assign structural features to the functional and catalytic properties of the human MAT proteins, and facilitate future studies to probe new catalytic and binding functions.

## INTRODUCTION

MAT (methionine adenosyltransferase, also known as *S*-adenosylmethionine synthetase; EC 2.5.1.6) is the only known enzyme that synthesizes SAM (*S*-adenosylmethionine), the principle methyl group donor and precursor for polyamine and glutathione synthesis [1,2]. MAT catalyses the transfer of the adenosyl group from ATP to the sulfur atom of Met (L-methionine), in an unusual two-step reaction cleaving at both ends of the ATP triphosphate chain [3]. First, the methionine sulfur attacks the ATP C5′ atom, yielding the intermediate PPP_i_ (tripolyphosphate) and product SAM. Secondly, hydrolysis of PPP_i_ produces PP_i_ (pyrophosphate) and P_i_ (orthophosphate), before all three products (SAM, PP_i_ and P_i_) are released. MAT constitutes the first reaction step of the essential ‘methionine cycle’, which serves to maintain the intracellular balance of methionine and its associated activities including protein synthesis, one-carbon transfer metabolism and transsulfuration [4].

MAT is present in almost all living organisms from bacteria to mammals, many of which have more than one isoforms. Mammals contain two MAT genes, *mat1a* and *mat2a*, encoding three catalytic isoforms [2]. The MAT1A gene product (MAT1A, MAT2A and MAT2B are also known in the literature as MAT subunits α1, α2 and β respectively) is expressed in adult liver, where most transmethylation reactions take place, and organizes into tetramer (isoform I) or dimer (isoform III). The MAT2A gene product, which forms dimers (isoform II), is expressed in fetal liver, non-hepatic tissues, and during rapid growth and de-differentiation. The three isoforms differ in kinetic properties, thereby providing a means to regulate the steady-state intracellular level of SAM, a direct determinant of cell growth [5]. The function of MAT2A is further dependent upon an interaction with the auxiliary subunit MAT2B, encoded by the *MAT2B* gene [6]. MAT2B is present only in mammals and bears no sequence homology with the catalytic MAT1A/2A subunits, but instead harbours signature motifs of the SDR (short-chain dehydrogenase/reductase) superfamily [7]. MAT2B was proposed to modulate the catalytic activity of MAT2A by lowering its *K*_m_ value for its substrate Met and the *K*_i_ value for its product SAM [8]. Two spliced variants of MAT2B have been reported, both of which are found in the nucleus and interact with the mRNA-binding protein HuR [9]. A nuclear function for MAT2B is also supported by the recent identification of MAT2A and MAT2B as part of a transcription co-repressor complex for the oncoprotein MafK [10].

Dysregulation of MAT1A, MAT2A and MAT2B has been correlated with disease pathology. Mutations on the human *MAT1A* gene have been linked to hepatic MAT deficiency (OMIM number 250850), an inborn error of methionine metabolism in which patients presented with isolated persistent hypermethioninaemia [11,12]. MAT enzymes also play a role in liver diseases, such as hepatic cirrhosis and HCC (hepatocellular carcinoma). In HCC, MAT gene expression is switched from *MAT1A* to *MAT2A*, accompanied by an induction of MAT2A and MAT2B protein levels [5,13,14]. This switch in MAT gene expression is proposed to facilitate the rapid cell growth and provide a proliferative advantage for cancer [15], and also makes MAT2A/MAT2B an attractive target for chemoprevention and treatment of HCC [16].

At the protein level, the catalytic isoforms of MAT eMAT (*Escherichia coli* MAT) [17] and rMAT (rat MAT) 1A [18] have previously been crystallized in various liganded complexes (see Supplementary Table S1 at http://www.biochemj.org/bj/452/bj4520027add.htm), revealing different binding modes with regard to the adenine ring of substrate ATP and product SAM in the active-site pocket. On the other hand, no structural information is as yet available for the MAT2B protein. To address some of the unresolved questions from a structural perspective, we determined the crystal structures of all catalytic (MAT1A and MAT2A) and regulatory (MAT2B) subunits for the hMAT (human MAT) family. The results of the present study provide the first atomic view of the eukaryotic MAT2B architecture, allow a comparison of the MAT1A/MAT2A active site with structural homologues and establish a molecular basis for MAT disease mutations.

## EXPERIMENTAL

### Cloning, expression and purification of the hMAT proteins

DNA fragments encoding hMAT1A (residues 16–395; NCBI GI number 4557737), hMAT2A (residues 1–395; NCBI GI number 5174529) and hMAT2B (residues 14–323; NCBI GI number 37182512) were subcloned into the pNIC28-Bsa4 vector incorporating an N-terminal TEV (tobacco etch virus)-cleavable His_6_ tag. The resulting plasmids were transformed into *E. coli* BL21(DE3) cells, cultured in Terrific Broth at 37°C until a *D*_600_ of ~1.5 was reached and induced with 0.5 mM IPTG (isopropyl β-d-thiogalactopyranoside) overnight growth at 18°C. Cells were harvested and homogenized in buffer A {50 mM Hepes (pH 7.5), 500 mM NaCl, 5 mM imidazole, 0.5 mM TCEP [tris-(2-carboxyethyl)phosphine] and EDTA-free protease inhibitor}. Insoluble debris was removed by further centrifugation.

Proteins were purified by passing cell extracts through Ni-NTA (Ni^2+^-nitrilotriacetate) resin pre-equilibrated with buffer A and eluted with buffer B (buffer A and 250 mM imidazole). The eluted fractions were applied on to a HiLoad 16/60 Superdex 200 column pre-equilibrated with GF buffer [10 mM Hepes (pH 7.5), 500 mM NaCl, 5% glycerol and 0.5 mM TCEP]. For hMAT1A, the eluted fractions were diluted to a [NaCl] of 50 mM, and applied to a 5 ml HiTrap HP-Q column pre-equilibrated with IEX buffer [20 mM Tris/HCl (pH 8.0) and 50 mM NaCl]. Protein was eluted with a linear NaCl gradient (0–1 M). For hMAT2A and hMAT2B, the eluted proteins from the Superdex 200 column were treated with TEV protease overnight at 4°C and passed over Ni-Sepharose resin pre-equilibrated with GF buffer. The final purified proteins were concentrated to 16 mg/ml (hMAT1A), 19 mg/ml (hMAT2A) and 25 mg/ml (hMAT2B) and stored at −80°C.

### Crystallization and structure determination of hMAT1A and hMAT2A

Crystals were grown by the sitting-drop vapour diffusion method. Prior to crystallization, 5 mM SAM was added to both proteins. Crystals were cryoprotected and flash-frozen in liquid nitrogen. Diffraction data were collected at the Swiss Light Source beamline X10SA and processed with the HKL package [19]. Both hMAT1A and hMAT2A crystallized in the orthorhombic space group *I*222 with one molecule in the asymmetric unit. The structure of hMAT2A was first solved by molecular replacement using the program PHASER [20] with the structure of rMAT1A (PDB code 1QM4) as a search model. Iterative cycles of restrained refinement and manual model building were performed using COOT [21] and REFMAC5 [22]. The structure of hMAT1A was subsequently solved by molecular replacement with the refined MAT2A structure. Calculation of difference Fourier maps revealed clear electron density for SAM in the active sites for hMAT1A and hMAT2A.

### Crystallization and structure determination of hMAT2B_subt_

Purified hMAT2B was digested with subtilisin for 1 h at 293 K at a protein/subtilisin mass ratio of 1:30. At selected time points, 10 μl aliquots were removed and the reaction was quenched by the addition of 1 μl of 100 mM PMSF, before SDS/PAGE analysis. Crystals were grown by vapour diffusion in sitting drops at 4°C. A sitting drop consisting of 100 nl of protein (hMAT2B_subt_) and 200 nl of well solution was equilibrated against well solution containing 1.05 M lithium sulfate and 0.45 M TMAO (trimethylamine *N*-oxide). The crystals were mounted directly from the drop using 25% ethylene glycol as a cryoprotectant and flash-frozen in liquid nitrogen. A two-wavelength dataset was collected at the Diamond Light Source (Harwell, U.K.; Table 1) and processed using the CCP4 suite [23]. Phases were obtained by selenium–MAD phasing using SHELXD [24] which identified five selenium sites per monomeric protein. The sites were used for phasing with SHELXE and optimized in SHARP/AutoSHARP [25]. The initial model was built using ARP/wARP [26] followed by iterative cycles of manual building in COOT [21] and refinement with REFMAC5 [22].

**Table 1:** Summary of the data collection and refinement statistics The values in parentheses are for the highest resolution shell.

Parameter	hMAT1A	hMAT2A	hMAT2B_resv_	hMAT2B_subt_ (λ_1_)	hMAT2B_subt_ (λ_2_)
Data collection					
Space group	*I*222	*I*222	*P*422_1_2	*C*222_1_	*C*222_1_
Wavelength (Å)				0.980	0.984
a, b, c (α=β=γ=90^o^)	66.40, 95.01, 115.77	68.30, 94.11, 117.38	163.39, 163.39, 252.88	41.02, 111.70, 123.11	41.05, 111.85, 123.22
Resolution range (Å)	57.83–1.90	73.52–1.21	58.96–2.8	61.55–2.68	61.61–2.17
Number of unique reflections	27355	113895	78936	7705	15419
*R*_merge_ (%)	0.11 (0.22)	0.05 (0.19)	0.05 (1.03)	0.06 (0.31)	0.05 (0.91)
<*I*>/<σ*I*>	9.7 (5.1)	23.1 (10.93)	9.8 (1.1)	8.6 (2.4)	9.6 (2.4)
Completeness (%)	93.7 (68.4)	99.0 (97.5)	93.2 (56)	92.8 (64.1)	99.8 (98.9)
Multiplicity	3.8 (2.2)	7.16 (6.48)	6.9 (2.9)	5.7 (3.1)	6.6 (6.3)
Anomalous completeness (%)				87.0	99.3
Anomalous redundancy				3.1	3.4
Refinement					
Maximum resolution used (Å)	2.05	1.21	2.80		2.25
Number of reflections	21754	108185	78848		12589
*R* factor (%)	0.141	0.101	0.169		0.177
Free *R* factor (%)	0.183	0.119	0.190		0.218
RMSD bond lengths (Å)	1.383	1.615	1.110		0.970
RMSD bond angles (^o^)	0.013	0.013	0.010		0.009
PDB accession code	2OBV	2P02	2YDX		2YDY

### Crystallization and structure determination of hMAT2B_resv_

Prior to crystallization, 5 mM NADP and 2 mM resveratrol were added to the protein. Crystals were grown by vapour diffusion in sitting drops at 4°C. A sitting drop consisting of 100 nl of protein (hMAT2B_resv_) and 200 nl of well solution was equilibrated against well solution containing 1.05 M lithium sulfate and 0.45 M TMAO. The structure of hMAT2B_resv_ was determined by molecular replacement in PHASER [20] using the refined hMAT2B_subt_ structure as search model. The structure was refined with alternating cycles of restrained refinement in BUSTER (http://www.globalphasing.com/buster/) and manual building in COOT [21]. In the final stages of refinement, atomic displacement parameters were refined with TLS (Translation–Libration–Screw-rotation) parameters.

### DSF (differential scanning fluorimetry)

hMAT2B was assayed for shifts in melting temperature caused by the presence of various ligands in a 96-well PCR plate using an Mx3005p RT (real-time)-PCR machine (Stratagene). Each well (25 μl) consists of protein (2.5 μM in buffer A), SYPRO-Orange (Invitrogen) diluted ×1000 and 1 mM nucleotide/ligand. Fluorescence intensities were measured from 25°C to 96°C with a ramp rate of 1°C/min. The temperature shifts, Δ*T*_m_^obs^, for each ligand were determined as described previously [28].

## RESULTS AND DISCUSSION

### Structural insights into catalytic mechanism of hMAT1A and hMAT2A

**Figure 1:**
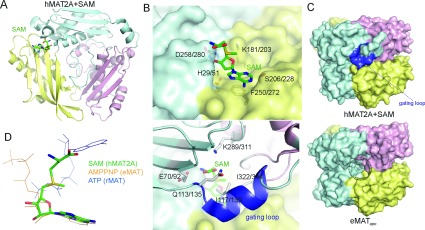
Structures of hMAT1A and hMAT2A (**A**) Structure of the hMAT2A monomer, coloured yellow for the N-domain, cyan for the central domain and pink for the C-domain. The bound product SAM is shown as sticks. (**B**) Upper panel, the interactions of SAM with the ‘lower half’ of the binding pocket formed by one subunit of the dimer (in surface representation). Lower panel, interactions of SAM with the ‘upper half’ of the binding pocket formed by the second subunit of the dimer (in ribbons). (**C**) Surface representation of the eMAT (apo form, PDB code 1XRA) and hMAT2A dimers, emphasizing the ordered gating loop in hMAT2A (coloured blue) which blocks access to the active site. (**D**) Superimposition of ATP, AMP-PNP and SAM from the structures of rMAT (PDB code 1O9T), eMAT (1P7L) and hMAT2A (the present study) respectively.

The structures of hMAT1A and hMAT2A are nearly identical to each other [RMSD (root mean square deviation) 0.41 Å (1 Å=0.1 nm)] and similar to the reported structures of rMAT1A and eMAT (RMSD ~1.55 Å and 2.24 Å respectively), all adopting a three-domain architecture (Figure 1A). In the crystals, hMAT1A and hMAT2A exist as dimer of dimers (see Supplementary Figure S1A at http://www.biochemj.org/bj/452/bj4520027add.htm), similar to the tetrameric packing of eMAT and rMAT1A with minor differences in the interdimer orientation [18,29]. Each dimer consists of two active sites located at the subunit interface. Key active-site residues in eMAT [30] are conserved in the human (see Supplementary Figure S2 at http://www.biochemj.org/bj/452/bj4520027add.htm), including His^29/51^ (residue numbering hereafter denotes hMAT1A/hMAT2A) from the HPDK motif that functions as an acid/base catalyst in the SAM formation step, as well as several basic residues (Arg^264/286^, Arg^265/287^ and Lys^285/307^) that orientate the PPP_i_ moiety in the hydrolysis step.

The hMAT1A/hMAT2A structures reveal a bound SAM molecule at each active site, sandwiched at the dimer interface. SAM adopts a bent conformation with the adenine and ribose moieties fitting into one subunit of the dimer (Figure 1B, upper panel) and the methionine group protruding into the opposite subunit of the dimer (Figure 1B, lower panel). The binding of SAM is partially stabilized by a surface-exposed ‘gating loop’ (residues 113–122/135–144) that exhibits sequence variability across species (see Supplementary Figure S2). The gating loop is disordered in many unliganded structures rendering the active site solvent-accessible (Figure 1C, lower panel). In our SAM-bound structures, the gating loop adopts an ordered conformation which is also seen in the eMAT structures bound with both substrates {e.g. AMP-PNP (adenosine 5′-[β,γ-imido]triphosphate) and Met} or both products [PNPP (*p*-nitrophenyl phosphate) and SAM] (see Supplementary Table S1) [17], such that access to the active site is obstructed (Figure 1C, upper panel). The hMAT1A/hMAT2A structures suggest that SAM alone is sufficient to render the gating loop ordered, possibly owing to the presence of the methionine moiety. The gating loop therefore can assume a direct role in catalysis, to sense the presence and proper orientation of a methionine moiety in either the substrate Met or product SAM, and to ensure the correct configuration of substrates for favourable nucleophilic attack.

The adenine ring and ribose moieties of SAM in the hMAT1A/hMAT2A active site superimpose well with those of AMP-PNP (substrate analogue) or SAM (product) bound in the reported eMAT structures [17]. The human and *E. coli* data corroborate with the hypothesis that the enzyme substrates (ATP and Met) and products (SAM and PPP_i_) can occupy the active site in similar orientations (with regard to the adenosine moiety) and engage in similar protein-binding interactions. The rMAT1A structures bound with substrate, product or analogue, however, reveal a different orientation of the adenine ring and large distances (>9 Å) separating the reactive methionine sulfur and ATP C5′ atoms [18]. The plethora of ligand-bound MAT structures available to date (summarized in Supplementary Table S1) may therefore represent the different possible ligand conformations, potentially mediated by the nearby protein gating loop, that the substrates and products could adopt within the active site during different stages of catalysis. Such variety of ‘intermediate’ structural snapshots also highlights the dynamics and flexibility within the conserved MAT active site, and merits further structural and biochemical studies to delineate the underlying complexity of the catalytic mechanism.

### A structural rationale for the hMAT1A disease mutations

**Figure 2:**
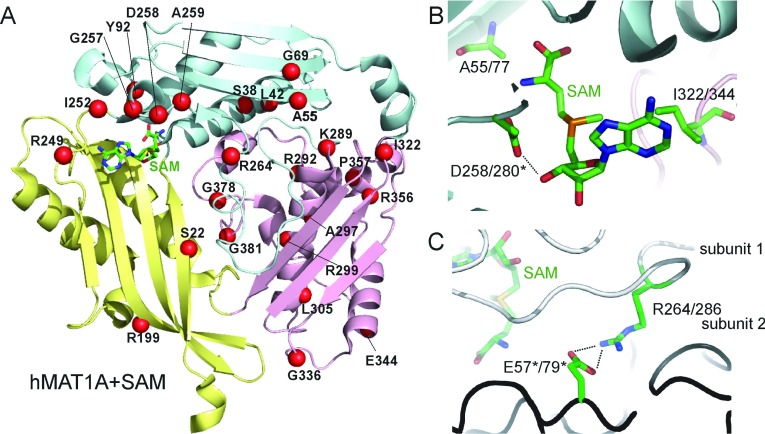
Structural basis for hMAT1A deficiency (**A**) Ribbon diagram of the hMAT1A, highlighting the sites of pathogenic missense mutations (red spheres) causing hepatic hMAT1A deficiency. (**B**) A close-up view of the SAM-binding pocket showing locations of the missense mutations A55D, D258G and I322M. (**C**) A close-up view of the dimer interface showing the salt bridge between Arg^264/286^ of one subunit and Glu^57/79^ of another subunit of the dimer. In (**B**) and (**C**) the residue numbering of hMAT1A/hMAT2A is shown. Asterisks denote residues from the adjacent subunit of the dimer.

To establish a structural basis for inherited MAT1A deficiency, we mapped the reported pathogenic mutations, including 30 missense mutations (see Supplementary Table S2 at http://www.biochemj.org/bj/452/bj4520027add.htm), in the hMAT1A structure. The nonsense and indel mutations are expected to cause premature translation termination, resulting in truncated proteins that affect enzyme function and stability. The 30 missense mutations are located along the entire hMAT1A polypeptide, albeit with a preponderance in the second and third subdomains (Figure 2A, red spheres). The mutation sites are found to be conserved in both hMAT1A and hMAT2A sequences (see Supplementary Figure S2), and their biochemical and structural penalties can be interpreted with regard to three categories: mutations that affect the (i) active-site binding pocket, (ii) oligomerization interface, and (iii) overall stability of the structure.

The first category consists of amino acid changes at the active site, including A55D, G69S, D258G and I322M/V. These residues line the SAM-binding pocket and form hydrogen bonds (e.g. Asp^258^) and van der Waals forces (e.g. Ala^55^ and Ile^322^) with the SAM ligand (Figure 2B). Therefore introduction of bulkier and longer side-chains or removal of polar contact atoms would affect the shape complementarity of the pocket and consequentially reduce the SAM-binding affinity. These mutations were found to reduce enzyme activity by >50% [31,32]. The second category of missense mutations is found at the dimer interface, e.g. R249W, I252T, G257R, A259V and R264C/H. In particular Arg^264^ is involved in a salt bridge with Glu^57^ from the dimeric partner (Figure 2C). Its substitution to a cysteine or histidine residue almost completely abolished enzyme activity [31,33], which may be owing to the weakened ability of the mutant to dimerize. The third category of mutations is distributed across the entire polypeptide and may destabilize the overall protein fold by means of steric hindrance with a bulky residue (S22L, S38N, A297D, P357L and G381R), early termination of helical structure (L42P and L305P), and the disruption of salt-bridge contacts (R199C, K289N, R292C, E344A and R356P/Q/W) and β-turns (G336R and G378S). The catalytic effects of these mutations vary in the range of 0.2–46% of the wild-type activity [32,34,35].

### Strategies to crystallize hMAT2B

**Figure 3:**
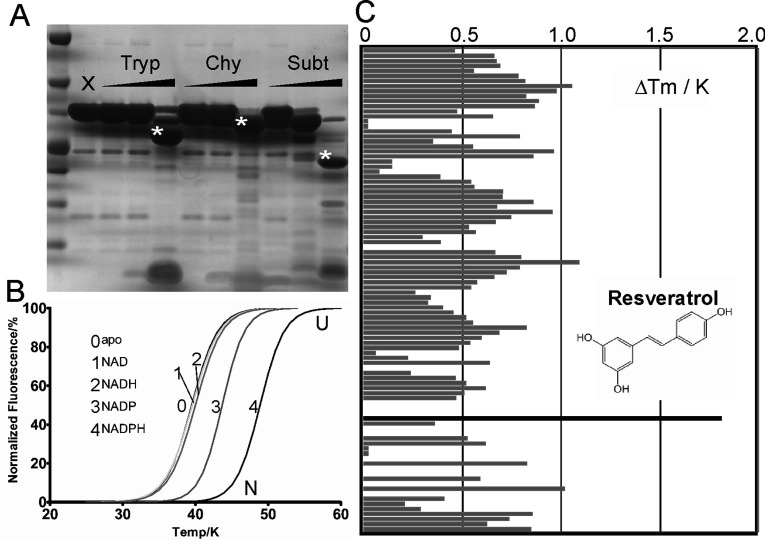
Remedial strategies to crystallize hMAT2B (**A**) Limited proteolysis of full-length hMAT2B (6 μg per reaction) in the presence of increasing quantity of trypsin (Tryp), chymotrypsin (Chy) and subtilisin (Subt). For each proteolytic enzyme, the three different amounts added are 200, 10 and 0.5 ng. A negative control of no proteolytic treatment (X) is also shown. Asterisks indicate stable truncated products. (**B**) Thermostability of hMAT2B in dependence of cofactor-bound states, as measured by DSF. The *T*_m_ value (°C) of each protein sample is the mid-point between the native (N) to unfolded (U) transition of the protein. (**C**) Stabilization of hMAT2B by a selection of small-molecule compounds screened in the presence of NADP, and the Δ*T*_m_ value (against the no compound reference) for each compound is shown as a bar on the *y*-axis.

We pursued a structural study on hMAT2B to further understand its functional and biochemical properties. Despite a large yield of recombinant hMAT2B protein from *E. coli* overexpression, our efforts to crystallize the full-length and truncation constructs of hMAT2B were not been successful. To circumvent this we carried out two remedial strategies, namely *in situ* limited proteolysis and co-crystallization with small ligands. Limited proteolysis is a valuable tool to remove disordered or flexible regions in purified proteins, and can assist in crystal lattice packing [36]. A small-scale treatment of purified hMAT2B with limited amount of trypsin, chymotrypsin or subtilisin generated stable truncated products (Figure 3A, asterisk). We subsequently incorporated into the large-scale purification protocol a limited proteolysis step followed by size-exclusion chromatography. The purified truncated polypeptides, found to have molecular masses of ~28 kDa (subtilisin-treated hMAT2B) and ~32 kDa (trypsin-treated hMAT2B) by MS, were subjected to crystallization trials. The subtilisin-treated protein (hMAT2B_subt_) yielded diffraction-quality crystals allowing its structure determination at 2.2 Å resolution in the unliganded form (Table 1). Inspection of the structure revealed that crystal packing is mediated by the removal of three surface-exposed flexible regions in the subtilisin-treated hMAT2B protein, namely Phe^60^–Ala^77^, Ala^95^–Asn^113^ and the C-terminal residues 325–338 (see Supplementary Figure S3 at http://www.biochemj.org/bj/452/bj4520027add.htm).

We also investigated the effect of small-molecule ligands to stabilize protein conformations using DSF as a means to aid crystallization [37]. DSF analysis of ligand binding is based on the concept that proteins are stabilized by their ligands, and the difference between the denaturation temperatures of the ligand-bound and ligand-free forms (i.e. Δ*T*_m_) can be measured. This approach is independent of protein function and allows us to identify ligands which may not have been predicted *a priori*. We first set out to identify whether hMAT2B binds the cofactors NAD(P)/H in its oxidized or reduced state, and observed that the thermal stability of MAT2B was increased with NADP/H (Δ*T*_m_ of 4.5/9.7°C), but there is little or no stabilization with NAD/H (0.4/0.8°C) (Figure 3B). We subsequently screened against hMAT2B a manually compiled library of >600 chemical compounds that are known SDR substrates, inhibitors or cofactor analogues, and identified resveratrol with a Δ*T*_m_ value of 1.8°C in the presence of NADP (Figure 3C). We subsequently co-crystallized hMAT2B with NADP and resveratrol and determined the ligand-bound structure (hMAT2B_resv_) at 2.8 Å resolution (Table 1).

### hMAT2B is an SDR protein with preference for NADP binding

**Figure 4:**
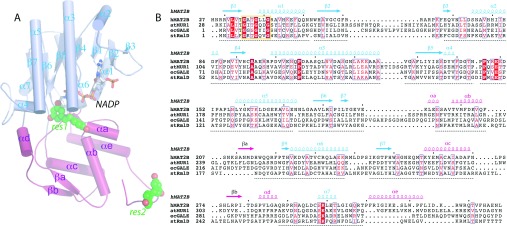
Structure of hMAT2B (**A**) The hMAT2B protomer is coloured in violet for the core Rossmann-like domain and magenta for the second extension domain. The bound NADP and resveratrol molecules (res1 and res2) are shown. (**B**) Structure-based sequence alignment of the NDP-sugar modifying subfamily of SDRs, including hMAT2B (PDB code 2YDX), *Arabidopsis thaliana* GDP-mannose-4,6-dehydratase (atMUR1; PDB code 1N7G), *E. coli* UDP-galactose-4-epimerase (ecGALE; PDB code 1A9Y) and *Salmonella typhimurium* dTDP-6-deoxy-lyxo-4-hexulose reductase (stRmlD; PDB code 1KC3). Secondary structure elements for hMAT2B are shown above the aligned sequences.

The hMAT2B_resv_ and hMAT2B_subt_ structures are highly similar to each other (RMSD <0.35 Å), and can be structurally divided into two domains constructed from discontinuous stretches of the polypeptide (Figure 4A). The core Rossmann-like domain (residues 27–183, 224–254 and 293–308) consists of a seven-stranded β-sheet sandwiched between six α-helices. It harbours the conserved signature sequences for SDRs [7], including the glycine-rich dinucleotide-binding motif (G^35^ATG^38^LLG^41^) for cofactor binding, as well as the catalytic triad (S^136^…..Y^159^GKTK^163^) which are important for substrate activation (Figure 4B). The second smaller domain (residues 184–223, 255–292 and 309–338) elaborates from the core Rossmann fold and contains five α-helices and a double-stranded β-sheet. Minor structural variations between the two structures are found in a loop region of the second domain (residues 282–289, loop C), forming an extended coil in the hMAT2B_resv_ structure and a two-turn helix in the MAT2B_subt_ structure. This region is important in shaping a putative substrate-binding pocket (see below).

**Figure 5:**
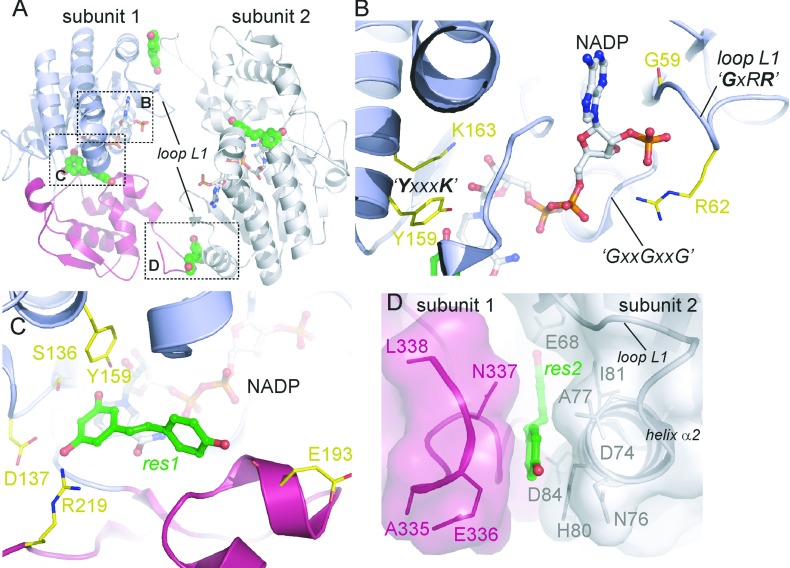
Ligand binding to hMAT2B (**A**) Structure of the hMAT2B dimer, bound with NADP (sticks) and resveratrol (spheres). (**B**–**D**) Enlarged views of ligand-binding sites of hMAT2B, revealing the binding modes of NADP in the core Rossmann-like domain (**B**), resveratrol molecule res1 close to the NADP (**C**) and resveratrol molecule res2 at the dimer interface (**D**). Subunit 1 of the dimer is coloured by domains, whereas subunit 2 is coloured grey.

Each hMAT2B_resv_ protomer is bound with an NADP molecule in its extended configuration (Figures 4A and 5A), packing against backbone atoms from the conserved GxxGxxG motif. The nicotinamide ribose hydroxyls are in hydrogen-bonding distances with Tyr^159^ and Lys^163^ from the YxxxK motif (Figure 5B). The adenosyl phosphate end of the cofactor is anchored by the loop segment L1 (connecting strands β2–α2) that contains the G^59^xRR^62^ sequence (Figure 5B). This L1 sequence is known to confer cofactor specificity for NADP in favour of NAD in many SDRs. In the case of hMAT2B, the preference for NADP over NAD can be explained by Gly^59^, which provides the space to accommodate the phosphate moiety, and Arg^62^, which forms hydrogen bonds to the phosphate oxygens (Figure 5B). The importance of loop L1 to NADP binding is also evidenced from the MAT2B_subt_ structure, in which the segment Phe^60^–Ala^77^ was not present in the structure (probably owing to subtilisin proteolysis) and no electron density for the NADP cofactor was observed despite being included in the crystallization buffer.

The MAT2B_resv_ structure reveals two bound resveratrol molecules (res1 and res2) per protomer, which exhibit different binding modes to the protein (Figure 5A, spheres). Res1 is positioned at the interdomain cleft of each monomer, in a putative ‘active site’ where most SDR enzymes bind their cognate substrates (Figure 5C). The benzene-1,3-diol group of res1 forms aromatic stacking with the NADP nicotinamide ring and the diol oxygens form hydrogen bonds with Ser^136^, Asp^137^, Tyr^159^ and Arg^219^. The hydroxystyryl group extends into a wide and shallow pocket in the second domain, with the hydroxyl oxygen atom hydrogen bonded to Glu^193^. Res2 slots into a narrow pocket that is formed by the C-terminal residues of one protomer (A), as well as the loop L1–helix α2 region of the adjacent NCS (non-crystallographic symmetry)-related protomer (B) within the asymmetric unit (Figure 5D). Protomers A and B form a head-to-tail dimer with an internal two-fold symmetry, contrasting with the typical back-to-back dimerization mode that involves a four-helical bundle as observed in many SDR oligomers. Res2 is positioned within the hMAT2B_resv_ dimer interface mainly by van der Waals contacts and hydrogen bonds with two acidic residues (Glu^68^ and Asp^84^).

### Structural homology of hMAT2B with nucleotide-sugar modifying enzymes

**Figure 6:**
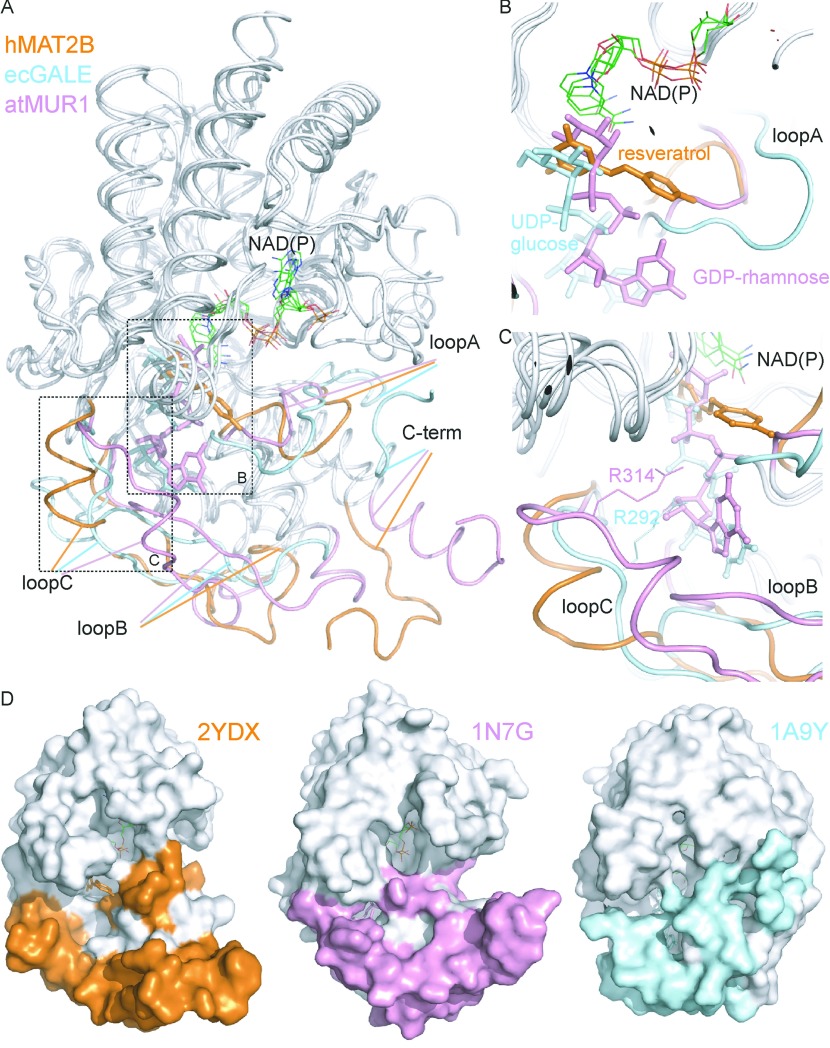
Structural superposition of NDP-sugar-modifying enzymes (**A**) Backbone superimposition of structural representatives from the NDP-sugar-modifying subfamily of SDRs, including hMAT2B (PDB code 2YDX), *A. thaliana* GDP-mannose-4,6-dehydratase (atMUR1; PDB code 1N7G) and *E. coli* UDP-galactose-4-epimerase (ecGALE; PDB code 1A9Y). (**B**) Enlarged views of the sugar-binding pockets in the second domain, the equivalent of which in hMAT2B is bound with resveratrol. (**C**) The arginine residues in *A. thaliana* GDP-mannose-4,6-dehydratase and *E. coli* UDP-galactose-4-epimerase that provide binding interactions to nucleotide sugar, residing in loop C, are not present in hMAT2B. (**D**) Surface representation of the three proteins highlighting the enlarged and solvent-accessible surface in the second domain of hMAT2B. (**A**–**C**) The three proteins are coloured in white except for the variable loop regions (loops A, B and C) of the second extended domain.

A DALI search [38] with hMAT2B identified a number of structural homologues from the SDR superfamily, including dTDP-rhamnose reductase (*Z*-score 33.7, sequence identity 29% and RMSD 2.1 Å; PDB code 1KC3) [39], UDP-glucose epimerase (*Z*-score 31.5, sequence identity 22%, and RMSD 2.5 Å; PDB code 1A9Y) [40], and GDP-mannose 4,6-dehydratase (*Z*-score 30.7, sequence identity 16% and RMSD 3.0 Å; PDB code 1N7G) [41]. These enzymes form a subclass of SDRs (Pfam accession number PF04321) that modify NDP-sugars in an NAD/P-dependent manner. The structures of the sugar-modifying homologues, bound with their respective cofactor and sugar substrates (Figure 6A), show that the substrate sugar moieties (e.g. rhamnose, mannose and glucose) are positioned in proximity to the cofactor, overlapping the binding region of resveratrol res1 in our hMAT2B_resv_ structure (Figure 6B). On the other hand, the NDP moieties (e.g. dTDP, UDP and GDP) are bound entirely within a narrow pocket in the smaller second domain (Figure 6C).

hMAT2B superimposes well with the sugar-modifying enzymes in the core Rossmann domain, but displays more structural differences in the second domain pocket, defined by loop C (residues 282–289), the parallel β-sheet preceding it (part of loop B; residues 210–214 and 275–279), as well as the loop preceding helix α_B_ (loop A; residues 199–206) (Figure 6A). These structure elements are oriented away from the core of hMAT2B, and form the platform of a broad and surface-exposed groove in hMAT2B (Figure 6D, left-hand panel). By contrast, the equivalent structure elements in other sugar-modifying enzymes are oriented more closely to the core, forming the lid that covers a narrower more secluded substrate pocket (Figure 6D, middle and right-hand panels). The binding determinant for the NDP diphosphate group in these enzymes, namely a conserved arginine residue near loop C (e.g. Arg^314^ in 1N7G and Arg^292^ in 1A9Y), is also absent in hMAT2B (Figure 6C). Together, these structural modifications of the hMAT2B second domain suggest against NDP-sugar binding for this protein and are consistent with our experimental observations that: (i) the DSF assay did not yield any detectable *T*_m_ shift with nucleotides (see Supplementary Figure S4A at http://www.biochemj.org/bj/452/bj4520027add.htm), and (ii) no naturally occurring sugar molecules derived from the expression host *E. coli* cells were co-purified, unlike in some sugar-modifying homologues [41].

A second distinguishing feature of hMAT2B concerns its oligomeric state. Size-exclusion chromatography with full-length hMAT2B reveals a monomeric species, as compared with the dimeric or tetrameric arrangements as observed in the sugar-modifying enzymes. The hMAT2B_resv_ structure did reveal a head-to-tail dimeric packing for the two NCS-related protomers within the asymmetric unit (Figure 5A), but it probably represents a crystallization artefact for the following reasons: (i) the C-terminus involved at the interface is partly derived from the vector-encoded sequence (AENL; residues 335–338), (ii) no equivalent dimerization is observed in the hMAT2B_subt_ crystal lattice, and (iii) size-exclusion chromatography of full-length hMAT2B does not reveal a dimeric species in solution (see Supplementary Figure S4B). Nevertheless, the hMAT2B_resv_ dimer interface, mediated by the res2 ligand, may mimick a physiologically relevant function in protein–protein interactions (see below).

### Concluding remarks

In the present study we performed a structural coverage for the hMAT family, which constitutes two catalytic isoforms and one accessory protein. The hMAT1A and hMAT2A isoenzymes are highly homologous at the structural level, not unexpected from their primary sequence conservation. As a result, the observed functional differences in their kinetic properties, physiological roles and disease relevance are probably the consequences of distinct subcellular localizations and differential regulation by accessory factors, such as the enigmatic MAT2B protein. Despite the discovery of the latter as a ‘MAT2A regulatory subunit’ for over a decade, its underlying function and mechanism remain unknown. The hMAT2B structure determined in the present study represents the first atomic view for this mammal-only protein, and confirms its membership within the highly ubiquitous SDR superfamily. Its structural homology with the sugar-modifying subclass of SDRs, however, is perhaps more intriguing and puzzling. It is currently unknown whether MAT2B performs similar NAD/P-dependent modifications on sugar substrates and, if so, what the functional relevance to the methionine cycle will be. The unambiguous presence of the catalytic triad (serine, tyrosine and lysine) in hMAT2B, residues known to stabilize SDR substrates for hydride transfer, nevertheless merits the search for potential substrates or binding ligands for hMAT2B, which is currently underway.

One established functional partner for MAT2B is the catalytic isoenzyme MAT2A, through which MAT2B is postulated to interact and modulate its catalytic efficiency in order to match the cellular requirement for SAM synthesis. There are precedents of SDR member enzymes functioning as regulatory proteins via protein–protein interactions, with examples including CtBP (C-terminal binding protein), HSCARG [also known as NMRAL1 (NmrA-like family domain containing 1)] and NmrA [42,43]. A unifying feature among these SDR regulators is their NAD(P)-sensing capability via the Rossmann fold. It is therefore not unreasonable to speculate that hMAT2B may also function as a redox sensor by dint of its NADP binding ability, as demonstrated in our structure and solution studies. This potentially allows hMAT2B to fine-tune hMAT2A catalytic activity to the cellular need and redox level of the cell. The manner in which MAT2A and MAT2B functionally associate to achieve the regulation is also unknown.

The structural elucidation of MAT2B, as well as the serendipitous identification of resveratrol as a small-molecule ligand, provides a starting framework to study its functional interactions. The two resveratrol-binding sites, one in the second domain ‘substrate pocket’ (res1 site) and the other at the crystal dimer interface (res2 site), may mimick possible contact regions for the MAT2A and MAT2B interactions. A number of surface-exposed loops are present within these two sites (e.g. loops A, B, C and L1), and exhibit limited sequence homology with other NDP-sugar-modifying enzymes, perhaps pointing to a specific role in protein–protein interaction. Pertinent to this, the equivalent regions (and their vicinity) in HSCARG and NmrA have been shown in biochemical and structural studies to be involved in direct interaction with their protein-binding partners (see Supplementary Figure S5 at http://www.biochemj.org/bj/452/bj4520027add.htm) [44,45]. With this in mind, the stage is therefore set for further experimental design aimed at isolating the MAT2A–MAT2B complex, in order to understand its molecular determinants and structural properties.

## Online data

Supplementary data
