# Supplementary material for: Insight into S-adenosylmethionine biosynthesis from the crystal structures of the human methionine adenosyltransferase catalytic and regulatory subunits
Source: Biochem J. 2013 Apr 25;452(Pt 1):27–36. doi: 10.1042/BJ20121580 (PMC3793261; doi:10.1042/BJ20121580)
Supplement: Supplementary data [file bj4520027add.pdf]

Biochem. J. (2013) 452, 27–36 (Printed in Great Britain) doi:10.1042/BJ20121580

SUPPLEMENTARY ONLINE DATA

Insight into *S*-adenosylmethionine biosynthesis from the crystal structures of the human methionine adenosyltransferase catalytic and regulatory subunits

Naeem SHAFQAT*, Joao R. C. MUNIZ*, Ewa S. PILKA*, Evangelos PAPAGRIGORIOU*, Frank VON DELFT*, Udo OPPERMANN*†[1] and Wyatt W. YUE*[1]

*Structural Genomics Consortium, University of Oxford, Oxford OX3 7DQ, U.K., and †Botnar Research Centre, NIHR Oxford Biomedical Research Unit, University of Oxford, Oxford OX3 7LD, U.K.

See the following pages for Supplementary Figures S1–S5 and Supplementary Tables S1 and S2.

[1] Correspondence may be addressed to either of these authors (email udo.oppermann@sgc.ox.ac.uk or wyatt.yue@sgc.ox.ac.uk).

The atomic co-ordinates and structure factors have been deposited in the PDB under accession codes 2OBV (hMAT1A), 2P02 (hMAT2A), 2YDY ($hMAT2B_{subt}$) and 2YDX ($hMAT2B_{resv}$).

© 2013 The Author(s)
The author(s) has paid for this article to be freely available under the terms of the Creative Commons Attribution Licence (CC-BY) (http://creativecommons.org/licenses/by/3.0/) which permits unrestricted use, distribution and reproduction in any medium, provided the original work is properly cited.

Biochemical Journal www.biochemj.org

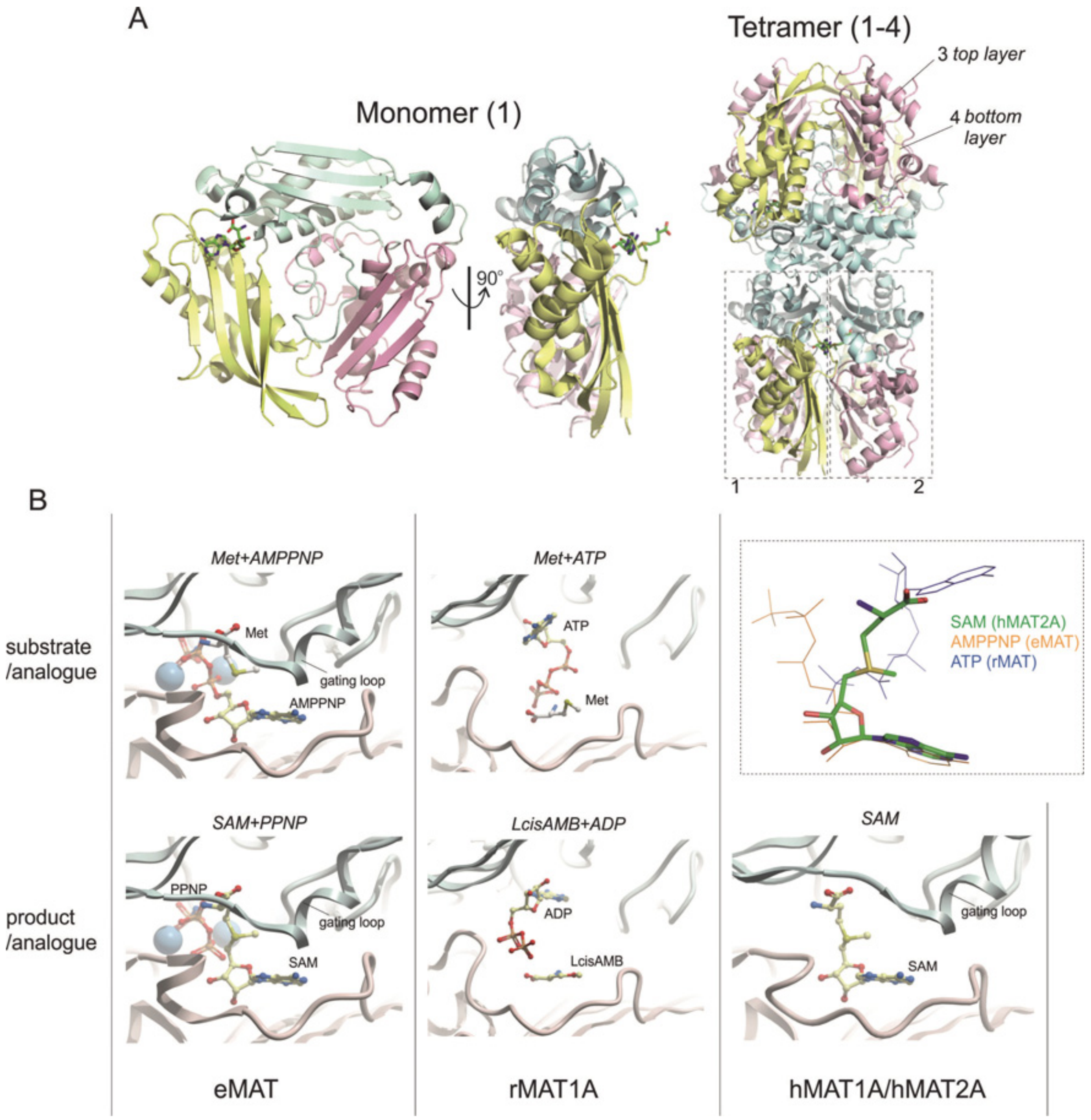

Figure S1 Crystal structures of hMAT1A/hMAT2A

(**A**) Left-hand panel, structure of the hMAT2A monomer in orthogonal views, coloured yellow for the N-domain, cyan for the central domain and pink for the C-domain. Right-hand panel, tetrameric assembly of hMAT2A showing two tightly packed dimers (1 and 2 and 3 and 4). In a dimer, the two active sites are located at the interface between two tightly packed monomers that are burying ∼20 % of the total accessible surface. Two dimers in turn form an elongated tetramer via less extensive interactions. (**B**) Substrate or product orientation in the active site. Interaction of substrates/analogues (upper panels) and products/analogues (lower panels) in the active-site pocket of eMAT, rMAT1A and hMAT1A/hMAT2A. The top subunit of the dimer is coloured cyan and the bottom subunit pink. The co-crystallized ligands from each structure are shown above the figure. Inset, superimposition of ATP, AMP-PNP and SAM from the structures of rMAT (PDB code 1O9T), eMAT (PDB code 1P7L) and hMAT2A (the present study) respectively.

© 2013 The Author(s)
The author(s) has paid for this article to be freely available under the terms of the Creative Commons Attribution Licence (CC-BY) (http://creativecommons.org/licenses/by/3.0/) which permits unrestricted use, distribution and reproduction in any medium, provided the original work is properly cited.

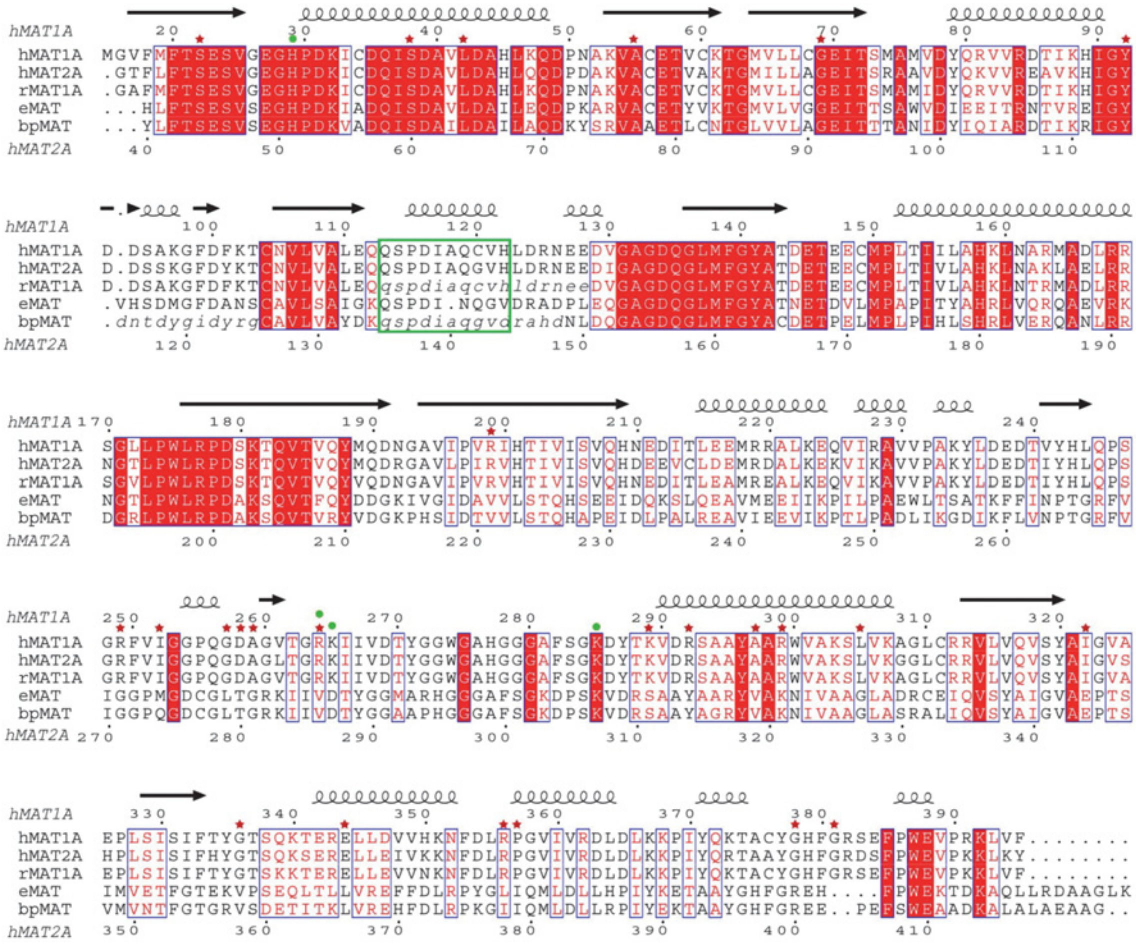

Figure S2 Structure-based sequence alignment of the MAT enzymes

The aligned sequences include hMAT1A (PDB code 2OBV; Uniprot ID Q00266), hMAT2A (PDB code 2P02; Uniprot ID P31153), rMAT1A (PDB code 1O9T; Uniprot ID P13444), eMAT (PDB code 1RG; Uniprot ID P0A817) and *Burkholderia pseudomallei* MAT (PDB code 3IML; Uniprot ID Q63YH5). Residue numbering and secondary structure elements for hMAT1A are shown above the aligned sequences. Residue numbering for hMAT2A is shown below the sequences. The conserved catalytic residues (Lys^{265}, Lys^{285}, Asp^{134}, Phe^{250} and His^{29}) mentioned in the main text are indicated in green circles. The gating loop is marked with green box. Residues with known clinical mutations in hMAT1A are marked with red stars. Residues that are not modelled in the crystal structures (presumably disordered) are shown in italics.

© 2013 The Author(s)
The author(s) has paid for this article to be freely available under the terms of the Creative Commons Attribution Licence (CC-BY) (http://creativecommons.org/licenses/by/3.0/) which permits unrestricted use, distribution and reproduction in any medium, provided the original work is properly cited.

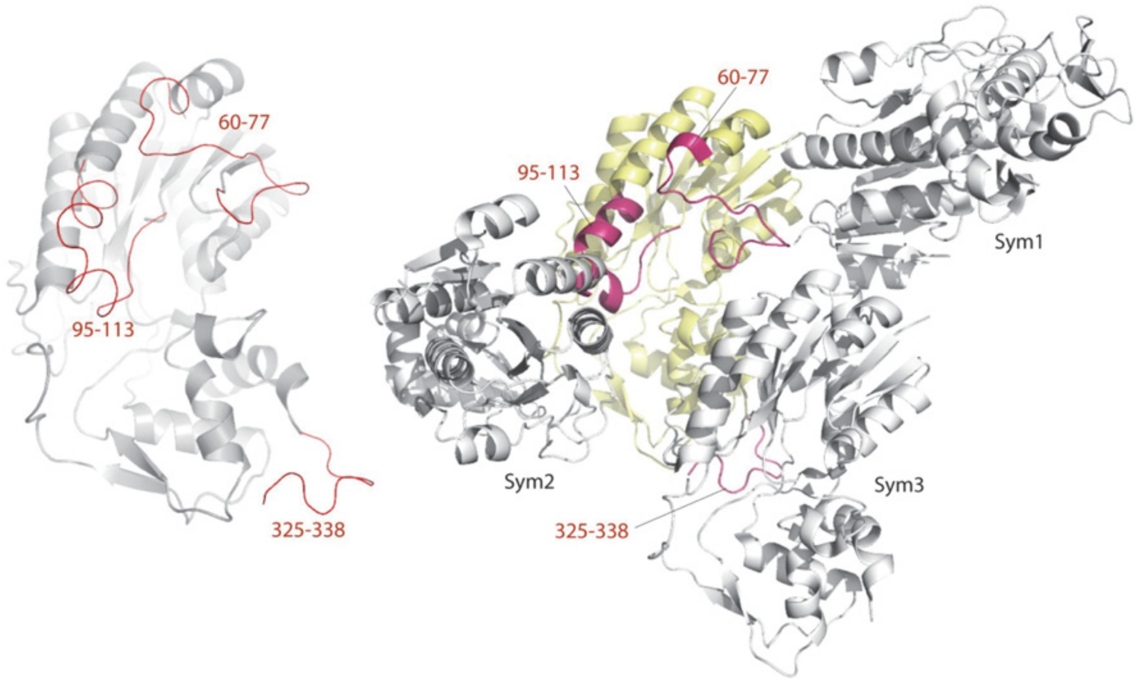

Figure S3 Crystal packing in the subtilisin-treated hMAT2B protein

Left-hand panel, the crystal structure of hMAT2B$_{subt}$ (grey) which reveals the absence of three loop regions (shown as an overlay from the hMAT2B$_{resv}$ structure; red). Right-hand panel, packing of crystallographic symmetry-related molecules (Sym1–3) of each hMAT2B$_{subt}$ protomer (yellow) was mediated by the absence of the three loop regions (shown as an overlay from the hMAT2B$_{resv}$ structure).

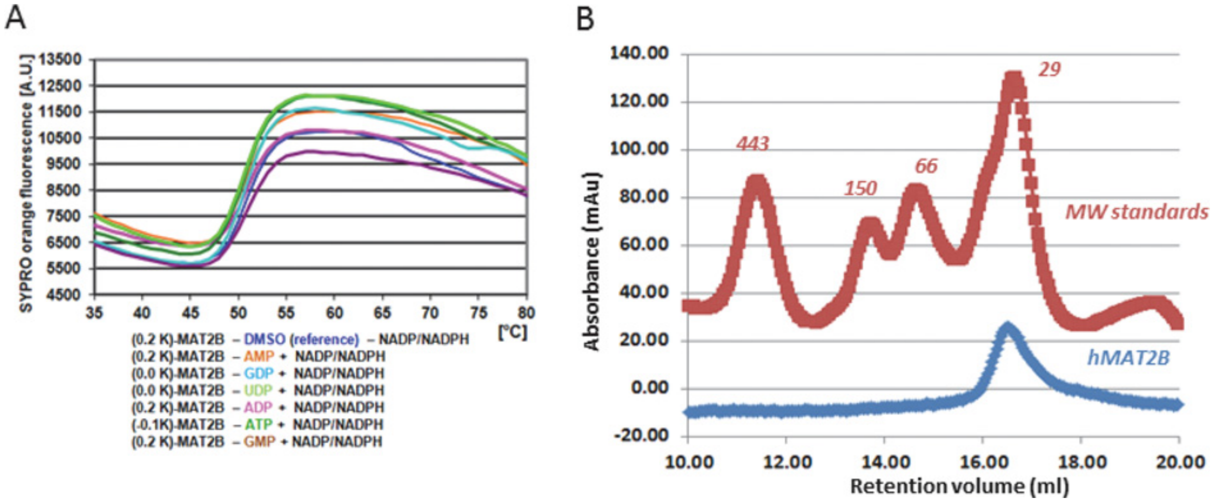

Figure S4 Solution studies of full-length hMAT2B

(**A**) Thermal stability assay of hMAT2B in the presence of various nucleotide sugars by DSF. (**B**) Size-exclusion chromatography of hMAT2B in solution, demonstrating a monomeric species. Elution peaks of molecular mass standards are shown in red.

© 2013 The Author(s)
The author(s) has paid for this article to be freely available under the terms of the Creative Commons Attribution Licence (CC-BY) (http://creativecommons.org/licenses/by/3.0/) which permits unrestricted use, distribution and reproduction in any medium, provided the original work is properly cited.

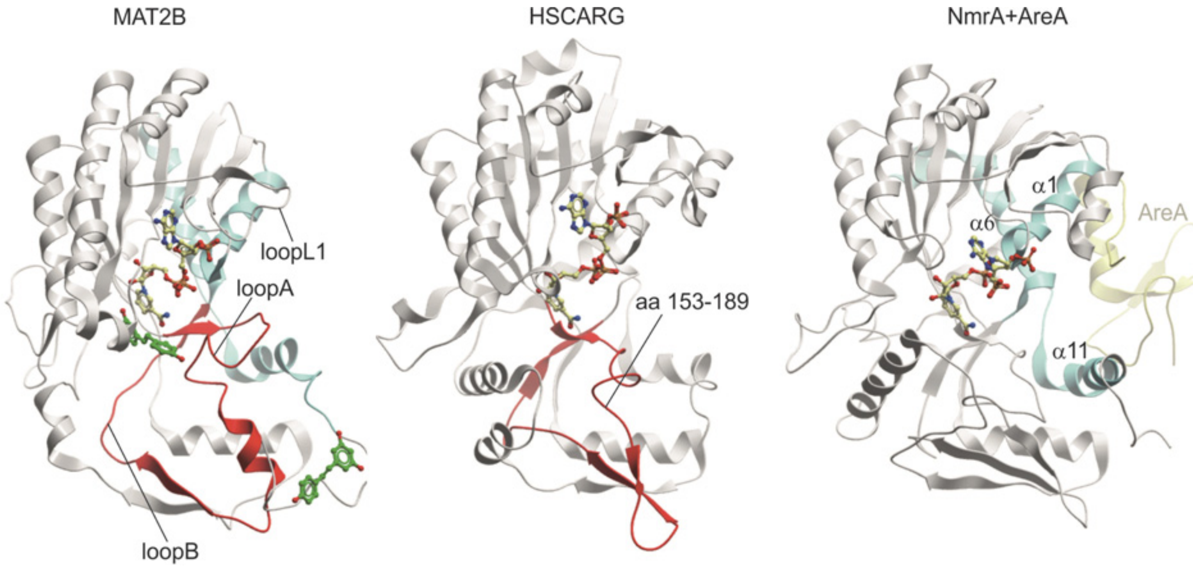

Figure S5 Mapping putative interaction sites on hMAT2B

The contact sites of two SDR homologues (red in HSCARG and cyan in NmrA) with their interaction partners [ASS1 (argininosuccinate synthase 1) and AreA respectively] are mapped on to the structure of hMAT2B. The PDB codes for the HSCARG–NADP and NmrA–NADP–AreA structures are 2EXX and 2VUU.

Table S1 Comparison of the MAT catalytic subunit structures determined to date

AEP, (2S,4S)-amino-4,5-epoxypentanoic acid; AMB, L-2-amino-4-methocy-cis-but-3-enoic acid; 8-Br-ADP, 8-bromo-ADP.

Organism	Name	PDB code	Resolution (Å)	Active site ligands	Gating loop*	References
Burkholderia pseudomallei		3IML	2.35		Disordered	Not published
Entamoeba histolytica		3SO4	3.18		Disordered	Not published
Mycobacterium marinum		3RV2	2.00		Disordered	Not published
Mycobacterium avium		3S82	1.73		Disordered	Not published
Mycobacterium tuberculosis		3TDE	1.85		Disordered	Not published
E. coli	eMAT	1P7L	2.50	AMP-PNP, Met	Ordered	[1]
		1RG9	2.50	SAM, PPNP	Ordered	[1]
		1FUG	3.20		Disordered	[2]
		1MXA	2.80	PP_i	Disordered	[3]
		1MXB	2.80	ADP	Disordered	[3]
		1MXC	3.00	8-Br-ADP	Disordered	[3]
		1XRA	3.00		Disordered	[4]
Rat	rMAT1A	1O90	3.10	AEP and PO_4	Disordered	[5]
		1O92	3.19	ADP and AMB and $3P_i$	Disordered	[5]
		1O93	3.49	ATP, AEP and $2P_i$	Disordered	[5]
		1O9T	2.90	ATP, Met and $2P_i$	Disordered	[5]
		1QM4	2.66	AMB and $2SO_4$	Disordered	[6]
Human	hMAT1A	2OBV	2.05	SAM	Ordered	The present study
	hMAT2A	2P02	1.21	SAM	Ordered	The present study

*Conformation of the gating loop as observed in the crystal structure

© 2013 The Author(s)
The author(s) has paid for this article to be freely available under the terms of the Creative Commons Attribution Licence (CC-BY) (http://creativecommons.org/licenses/by/3.0/) which permits unrestricted use, distribution and reproduction in any medium, provided the original work is properly cited.

Table S2 List of the clinical mutations identified for the hMAT1A gene

Activity is the percentage of mutant activity compared with the wild-type activity. n.r., not reported.

Amino acids	Nucleotides	Predicted consequence	Activity (%)	Reference(s)
Missense				
S22L	65C>T	Steric hindrance	~52	[7,8]
S38N	113G>A	Steric hindrance	None	[9]
L42P	125T>C	Disrupt helix	~10	[7,8]
A55D	164C>A	Disrupt SAM pocket	17.2	[10]
G69S	205G>A	Disrupt SAM pocket	~100	[8,11]
Y92H	274T>C	Disrupt polar interaction	~100	[8,12]
R199C	595C>T	Disrupt salt bridge	11.1	[13]
R249W	745C>T	Affect dimer interface	~18	[8,11]
I252T	755T>C	Affect dimer interface	~24	[8]
G257R	769G>A	Affect dimer interface	~10	[8]
D258G	773A>G	Disrupt SAM pocket	~5	[8]
A259V	776C>T	Affect dimer interface	~13	[8]
R264H	791G>A	Affect dimer interface	0.1	[9,14]
R264C	791C>T	Affect dimer interface	0.3	[9]
K289N	867G>T	Disrupt ionic interaction	~5	[8,11]
R292C	874C>T	Disrupt ionic interaction	~18	[13,15]
A297D	890C>A	Steric clash core	~25	[8]
R299H	896G>A	Disrupt ionic interaction	~17	[8]
R299C	895C>T	Disrupt ionic interaction	~25	[8]
L305P	914T>C	Disrupt helix	26	[10]
I322V	964A>G	Disrupt SAM pocket	~1	[8]
I322M	966T>G	Disrupt SAM pocket	45.8	[9]
G336R	1006G>A	Steric hindrance	22.9	[9]
E344A	1031A>C	Not known	12.1	[9]
R356P	1067G>C	Disrupt ionic interaction	~10	[8,11]
R356Q	1068G>A	Disrupt ionic interaction	53.1	[13]
R356W	1068C>T	Disrupt ionic interaction	~3	[8]
P357L	1070C>T	Steric hindrance	31	[10]
G378S	1132G>A	Disrupt β-turn	0.17	[13]
G381R	1141G>A	Steric hindrance	~30	[8]
Insertion				
185X	539insTG	Early termination	n.r	[13,16]
351X	827insG	Early termination	None	[13]
Deletion				
92X	255delCA	Early termination	None	[9]
350X	1043delTG	Early termination	n.r	[13]
Nonsense				
387X	1161G>A	Eight amino acid truncation	75	[17]

REFERENCES

1 Komoto, J., Yamada, T., Takata, Y., Markham, G. D. and Takusagawa, F. (2004) Crystal structure of the *S*-adenosylmethionine synthetase ternary complex: a novel catalytic mechanism of *S*-adenosylmethionine synthesis from ATP and Met. Biochemistry **43**, 1821–1831

2 Fu, Z., Hu, Y., Markham, G. D. and Takusagawa, F. (1996) Flexible loop in the structure of *S*-adenosylmethionine synthetase crystallized in the tetragonal modification. J. Biomol. Struct. Dyn. **13**, 727–739

3 Takusagawa, F., Kamitori, S. and Markham, G. D. (1996) Structure and function of *S*-adenosylmethionine synthetase: crystal structures of *S*-adenosylmethionine synthetase with ADP, BrADP, and PP_i at 28 angstroms resolution. Biochemistry **35**, 2586–2596

4 Takusagawa, F., Kamitori, S., Misaki, S. and Markham, G. D. (1996) Crystal structure of *S*-adenosylmethionine synthetase. J. Biol. Chem. **271**, 136–147

5 Gonzalez, B., Pajares, M. A., Hermoso, J. A., Guillerm, D., Guillerm, G. and Sanz-Aparicio, J. (2003) Crystal structures of methionine adenosyltransferase complexed with substrates and products reveal the methionine-ATP recognition and give insights into the catalytic mechanism. J. Mol. Biol. **331**, 407–416

6 Gonzalez, B., Pajares, M. A., Hermoso, J. A., Alvarez, L., Garrido, F., Sufrin, J. R. and Sanz-Aparicio, J. (2000) The crystal structure of tetrameric methionine adenosyltransferase from rat liver reveals the methionine-binding site. J. Mol. Biol. **300**, 363–375

7 Linnebank, M., Lagler, F., Muntau, A. C., Roschinger, W., Olgemoller, B., Fowler, B. and Koch, H. G. (2005) Methionine adenosyltransferase (MAT) I/III deficiency with concurrent hyperhomocysteinaemia: two novel cases. J. Inherit. Metab. Dis. **28**, 1167–1168

8 Fernandez-Irigoyen, J., Santamaria, E., Chien, Y. H., Hwu, W. L., Korman, S. H., Faghfoury, H., Schulze, A., Hoganson, G. E., Stabler, S. P., Allen, R. H. et al. (2010) Enzymatic activity of methionine adenosyltransferase variants identified in patients with persistent hypermethioninemia. Mol. Genet. Metab. **101**, 172–177

9 Chamberlin, M. E., Ubagai, T., Mudd, S. H., Thomas, J., Pao, V. Y., Nguyen, T. K., Levy, H. L., Greene, C., Freehauf, C. and Chou, J. Y. (2000) Methionine adenosyltransferase I/III deficiency: novel mutations and clinical variations. Am. J. Hum. Genet. **66**, 347–355

10 Ubagai, T., Lei, K. J., Huang, S., Mudd, S. H., Levy, H. L. and Chou, J. Y. (1995) Molecular mechanisms of an inborn error of methionine pathway. Methionine adenosyltransferase deficiency. J. Clin. Invest. **96**, 1943–1947

11 Chien, Y. H., Chiang, S. C., Huang, A. and Hwu, W. L. (2005) Spectrum of hypermethioninemia in neonatal screening. Early Hum. Dev. **81**, 529–533

12 Tada, H., Takanashi, J., Barkovich, A. J., Yamamoto, S. and Kohno, Y. (2004) Reversible white matter lesion in methionine adenosyltransferase I/III deficiency. AJNR Am. J. Neuroradiol. **25**, 1843–1845

13 Chamberlin, M. E., Ubagai, T., Mudd, S. H., Wilson, W. G., Leonard, J. V. and Chou, J. Y. (1996) Demyelination of the brain is associated with methionine adenosyltransferase I/III deficiency. J. Clin. Invest. **98**, 1021–1027

14 Nagao, M. and Oyanagi, K. (1997) Genetic analysis of isolated persistent hypermethioninemia with dominant inheritance. Acta Paediatr. Jpn. **39**, 601–606

15 Ito, M., Kotani, Y., Matsuda, J., Yokota, I., Naito, E., Mori, K. and Kuroda, Y. (2003) A methionine adenosyltransferase (MAT) deficiency patient treated with diet therapy. J Inherit Metab Dis. **26**, 76

16 Hazelwood, S., Bernardini, I., Shotelersuk, V., Tangerman, A., Guo, J., Mudd, H. and Gahl, W. A. (1998) Normal brain myelination in a patient homozygous for a mutation that encodes a severely truncated methionine adenosyltransferase I/III. Am. J. Med. Genet. **75**, 395–400

17 Kim, S. Z., Santamaria, E., Jeong, T. E., Levy, H. L., Mato, J. M., Corrales, F. J. and Mudd, S. H. (2002) Methionine adenosyltransferase I/III deficiency: two Korean compound heterozygous siblings with a novel mutation. J. Inherit. Metab. Dis. **25**, 661–671

Received 16 October 2012/11 February 2013; accepted 20 February 2013
Published as BJ Immediate Publication 20 February 2013, doi:10.1042/BJ20121580

© 2013 The Author(s)
The author(s) has paid for this article to be freely available under the terms of the Creative Commons Attribution Licence (CC-BY) (http://creativecommons.org/licenses/by/3.0/) which permits unrestricted use, distribution and reproduction in any medium, provided the original work is properly cited.
